# A QuEChERS-HPLC-MS/MS Method with Matrix Matching Calibration Strategy for Determination of Imidacloprid and Its Metabolites in *Procambarus clarkii* (Crayfish) Tissues

**DOI:** 10.3390/molecules26020274

**Published:** 2021-01-07

**Authors:** Qiuhong Yang, Xiaohui Ai, Jing Dong, Yongtao Liu, Shun Zhou, Yibin Yang, Ning Xu

**Affiliations:** 1 Yangtze River Fisheries Research Institute, Chinese Academy of Fishery Sciences, Wuhan 430223, China; dongjing@yfi.ac.cn (J.D.); liuyt@yfi.ac.cn (Y.L.); zhoushun@yfi.ac.cn (S.Z.); yangyb@yfi.ac.cn (Y.Y.); xuning@yfi.ac.cn (N.X.); 2 Chinese Academy of Fishery Sciences, Key Laboratory of Control of Quality and Safety for Aquatic Products, Ministry of Agriculture, Beijing 100141, China

**Keywords:** QuEChERS, HPLC-MS/MS, Imidacloprid, metabolites, 5-hydroxy imidacloprid, olefin imidacloprid, Imidacloprid urea, 6-chloronicotinic acid, *Procambarus clarkii*

## Abstract

We developed a method for determination of imidacloprid and its metabolites 5-hydroxy imidacloprid, olefin imidacloprid, imidacloprid urea and 6-chloronicotinic acid in *Procambarus clarkii* (crayfish) tissues using quick, easy, cheap, effective, rugged, and safe (QuEChERS) and high-performance liquid chromatography-triple quadrupole mass spectrometry. Samples (plasma, cephalothorax, hepatopancrea, gill, intestine, and muscle) were extracted with acetonitrile containing 0.1% acetic acid and cleaned up using a neutral alumina column containing a primary secondary amine. The prepared samples were separated using reverse phase chromatography and scanned in the positive and negative ion multiple reaction-monitoring modes. Under the optimum experimental conditions, spiked recoveries for these compounds in *P. clarkii* samples ranged from 80.6 to 112.7% with relative standard deviations of 4.2 to 12.6%. The limits of detection were 0.02–0.5 μg·L^−1^, the limits of quantification were 0.05–2.0 μg·L^−1^ and the method of quantification was 0.05–2.0 μg·kg^−1^. The method is rapid, simple, sensitive and suitable for rapid determination and analysis of imidacloprid and its metabolites in *P. clarkii* tissues.

## 1. Introduction

*Procambarus clarkii* is a species of freshwater crayfish native to northern Mexico and the southern and southeastern United States and has been introduced into many areas of China [1]. *P. clarkii* production has increased to over one million tons in 2018 leading to a commercial value of approximately RMB 369 billion total industrial output value in China [2]. In China, *P. clarkii* are primarily raised through integration into rice fields that makes their production more cost effective. However, this mode of cultivation also exposes these animals to pesticides and fertilizers used for rice cultivation and these types of effects have not been investigated [3].

Imidacloprid (1-6-chloro-3-pyridylmethyl-N-nitroimidazol-2-ylideneamine) is a neurotoxic insecticide of the neonicotinoid family class. It has high activity, broad insecticidal spectrum, good system physical properties and field stability. This compound is the current pesticide of choice to control sucking insect pests on rice, cotton, wheat, vegetables and fruit trees [4,5,6,7,8]. However, these pesticides are particularly dangerous because they are also toxic to beneficial insects such as honeybees [9,10]. Imidacloprid contains nitromethylene, nitroguanidine, cyanamidine and other pharma-codynamic groups. The neurotoxic substituent of imidacloprid is its nitroimine group [11,12,13]. The primary biodegradation products of imidacloprid include 5-hydroxy imidacloprid, olefin imidacloprid, imidacloprid urea and 6-chloronicotinic acid [3,14]. These bioconversions also alter the toxicity and olefin imidacloprid are 10–16 times more toxic than the parent compound [15]. These metabolites also retain insecticidal activity and metabolites derived through imidacloprid and olefin imidacloprid hydroxylation of nitroimine substituents are toxic to bees [16]. However, the toxicity to aquatic animals is unknown.

As a model breeding industry with an output revenue valued over RMB 100 billion, there was only one quality standard for crayfish in China at this time, which only stipulated seven kinds of veterinary drugs and one kind of heavy metal. With the increase of new pollutants, the change of cultivation mode and the upgrading of agricultural and veterinary drugs, these eight indicators are far from meeting the actual demand [17,18]. Therefore, to ensure the safety of crayfish used as human food, a detection method for imidacloprid and its metabolites must be established for *P. clarkii*.

Numerous detection methods have been established for imidacloprid and its metabolites in animal and plant tissues [14,19,20,21,22]. These have included measuring levels of imidacloprid in bees and honey products, pistachio nuts and green tea [12,16]. Interestingly, detection methods for imidacloprid and its metabolites in aquatic products have not been reported.

In the current study, we established an extraction and detection system to identify imidacloprid and its metabolites in *P. clarkii* tissues using QuEChERS (quick, easy, cheap, effective, rugged, and safe) and LC-MS (liquid chromatography-mass spectrometry). We then examined retail *P. clarkii* samples for the presence of imidacloprid and its metabolites.

## 2. Results and Discussion

### 2.1. Analyte Separation and Identification

**Figure 1:**
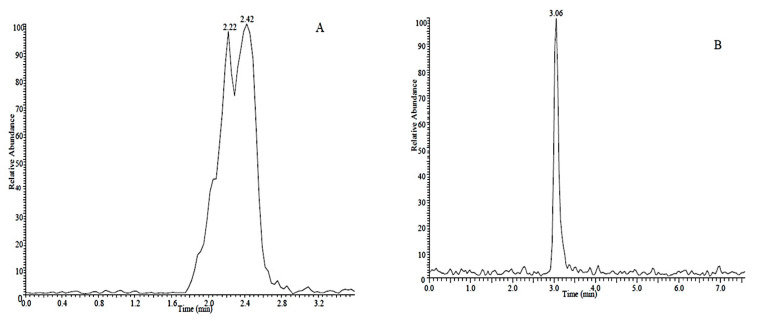
Chromatograms of 6-chloronicotinic acid using reverse phase LC and detection in negative ion mode in the (**A**) absence and (**B**) presence of 0.1% acetic acid as counterion.

We first optimized mass spectroscopic parameters for each individual target compound in positive and negative modes. Stock solutions (1 μg·mL^−1^) were injected into the ESI (electrospray ionization) source at a flow rate of 25 μL·min^−1^ and [M + H]^+^ molecular ion peaks for imidacloprid, imidacloprid-D4, 5-hydroxy imidacloprid and imidacloprid urea were established. In negative ion mode, the [M − H]^−^ molecular ion peaks were suitable for all compounds except 6-chloronicotinic acid that displayed a weak signal under these conditions. In order to improve the signal strength of 6-chloronicotinic acid, we connected a three-way valve to the ESI source, one side was into the standard solution, the other was into the mobile phase, which could adjust the pH and alter the signal strength. In the end, we found 6-chloronicotinic acid showed a better peak with an acidic mobile phase. Therefore, we acidified the solutions with 0.1% acetic acid to ensure 6-chloronicotinic acid eluted as one sharp peak in the LC chromatograms (Figure 1).

**Figure 2:**
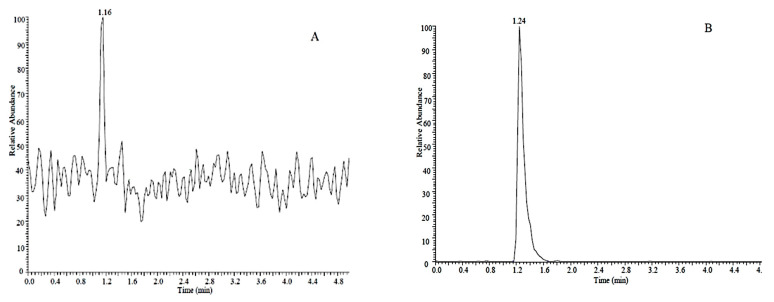
Olefin imidacloprid detection in (**A**) positive and (**B**) negative ion modes.

Olefin imidacloprid displayed high level detector responses in positive modes but the [M − H]^−^ molecular ion peak was greater and more symmetric in the negative ion mode (Figure 2).

**Figure 3:**
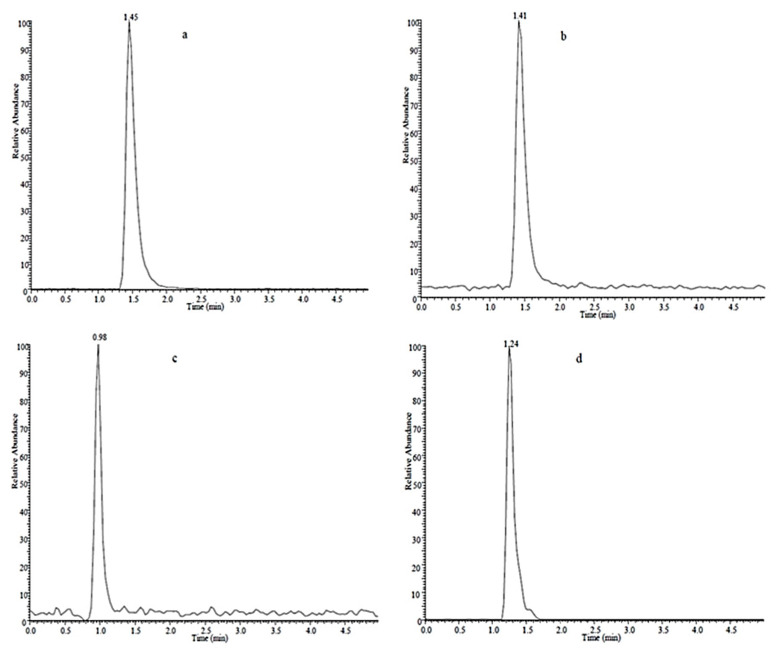

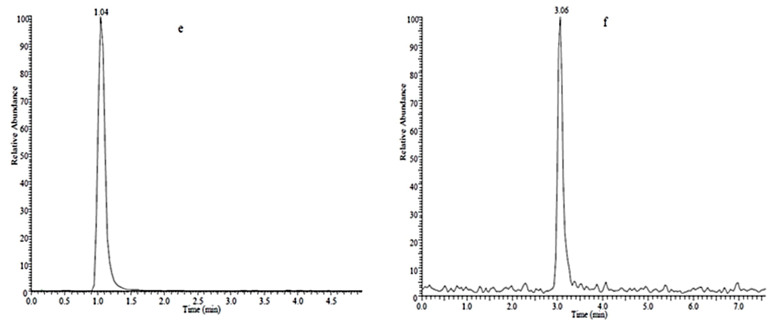
LC reverse phase separation of standard solutions of imidacloprid (**a**), imidacloprid-D4 (**b**), 5-hydroxy imidacloprid (**c**), olefin imidacloprid (**d**), imidacloprid urea (**e**) and 6-chloronicotinic acid (**f**).

We then optimized the chromatographic separations of imidacloprid and its target metabolites that were initially conducted using reverse phase chromatography with a methanol and water mobile phase in the presence and absence of 0.1% acetic acid as counterion. The inclusion of the counterion generated the most symmetrical peak shapes for these standards and was used for the remainder of the analytical separations (Figure 3).

### 2.2. Selection of Extraction Solvent

**Figure 4:**
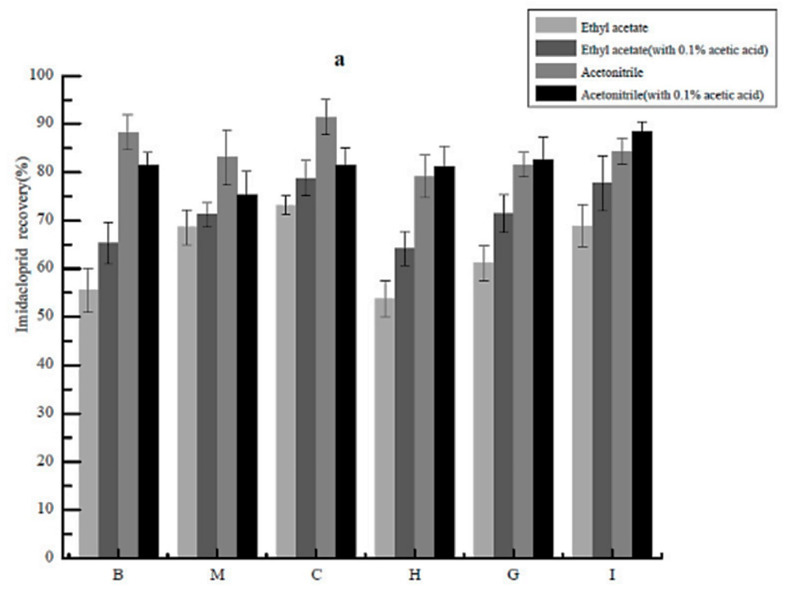

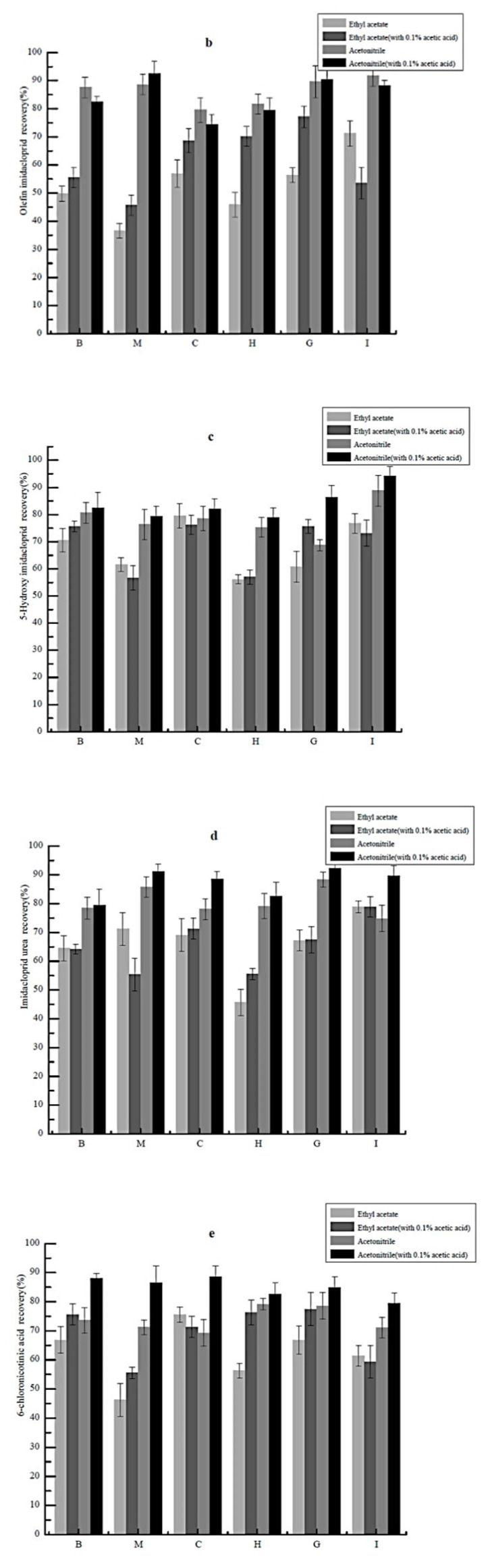
Recoveries of extractions of imidacloprid (**a**), olefin imidacloprid (**b**), 5-hydroxy imidacloprid (**c**), imidacloprid urea (**d**) and 6-chloronicotinic acid (**e**) using ethyl acetate and acetonitrile in the presence and absence of 0.1% acetic acid as indicated from the following *Procambarus clarkii* matrices: B, blood; M, muscle; C, cephalothorax; H, hepatopancrea; G, gills; I, intestine.

In order to improve the extraction efficiency and minimize matrix interference, the extraction solvent should have a polarity similar to the target compound [23]. Previous studies have indicated that acetonitrile and ethyl acetate possessed polarities similar to our target compounds and generated better recoveries, less interference from fats and proteins and less co-extracted matrix components [7,8,24,25]. Thus, we chose acetonitrile and ethyl acetate as the primary extraction solvents and examined extraction in the presence and absence of 1% acetic acid in *P. clarkii* tissue matrices. Overall, ethyl acetate gave lower recoveries than with acetonitrile and this was independent of acidification. The yields of 5-hydroxy imidacloprid, imidacloprid urea and 6-chloronicotinic acid in acetonitrile with 0.1% acetic acid solvents were >80% (Figure 4). Thus, acetonitrile containing 0.1% acetic acid was chosen as the extraction solvent. The addition of anhydrous MgSO_4_ and NaCl increased recoveries to 80–100%. The inclusion of these compounds most likely reduced the water phase and promoted partitioning of the pesticides into the organic layer as has been previously documented [26,27].

### 2.3. Optimization of Purification Conditions

**Figure 5:**
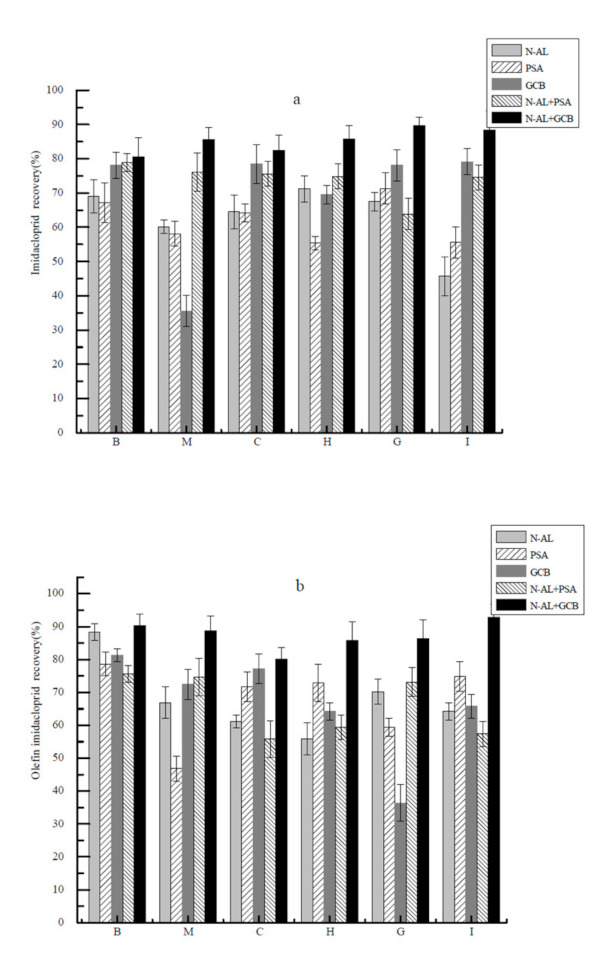

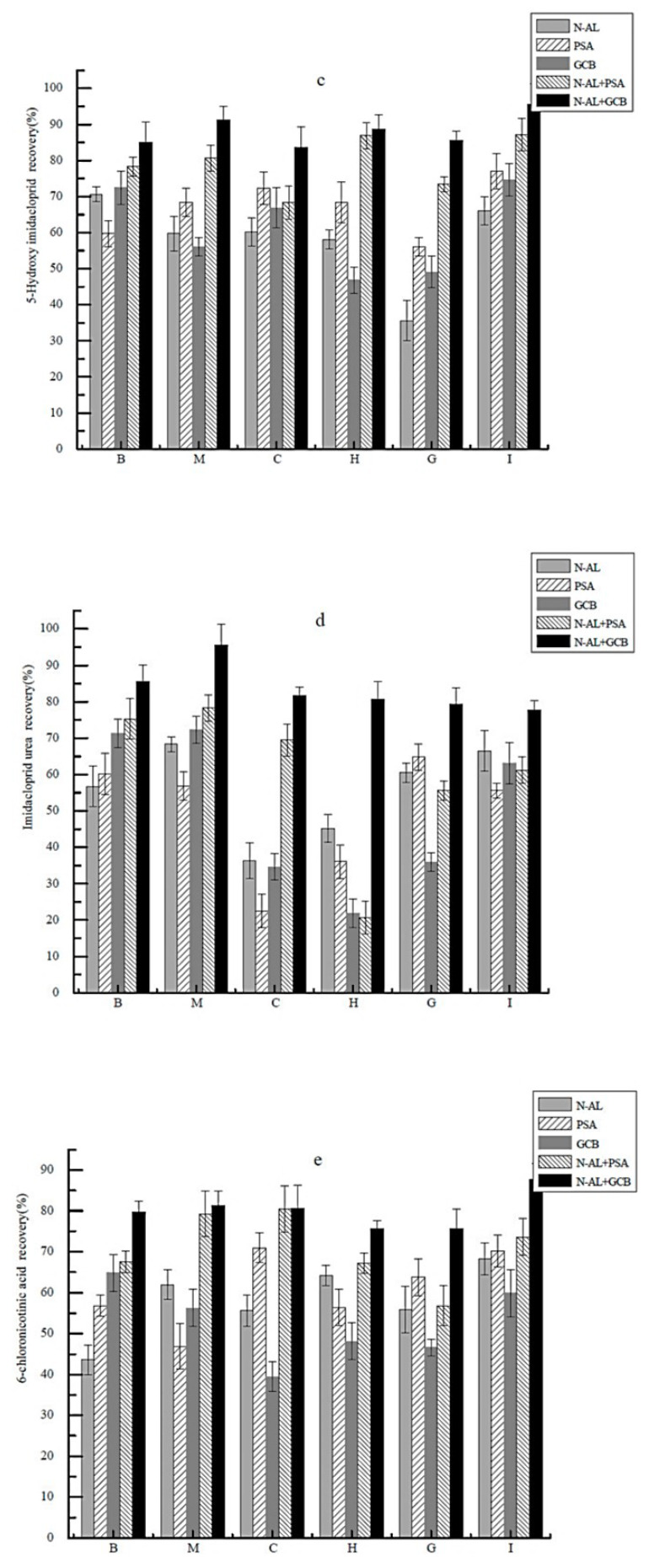
Effects of different purification materials on the recoveries of imidacloprid (**a**), olefin imidacloprid (**b**), 5-hydroxy imidacloprid (**c**), imidacloprid urea (**d**) and 6-chloronicotinic acid (**e**) from *P. clarkii* homogenates. PSA, primary secondary amine; GCB, graphitized carbon black; N-AL + PSA, neutral alumina and primary secondary amine; N-AL + GCB, neutral alumina and graphitized carbon black. See Figure 4 for abbreviations.

*P. clarkii* tissues represent a complex matrix and contain fats, protein, pigments and other substances [28]. We, therefore, developed a clean-up step using common chromatographic sorbents prior to LC injection. We examined the highly-polar neutral alumina that possesses properties close to silica gels that are commonly used to remove aromatic and aliphatic compounds [29,30]. PSA (primary secondary amine) is a weak anion exchanger that can remove fatty acids, sugars and other components that can form hydrogen bonds [31]. GCB (graphitized carbon black) can efficiently remove pigments especially chlorophyll [32,33]. Recoveries for imidacloprid, 5-hydroxy imidacloprid, olefin imidacloprid, imidacloprid urea and 6-chloronicotinic acid were all >80% for the neutral alumina column compared with the others. Moreover, this column extracted more interfering substances and achieved the best effect (Figure 5). GCB can effectively remove astaxanthin present in *P. clarkii*. As a result, the neutral alumina column combined with GCB was used for purification.

### 2.4. Linear Range, Matrix Effects, Detection Limits, Recoveries and RSD

Using the optimized conditions for separation and detection of imidacloprid and its metabolites, we performed a complete analysis of our test compounds using a series of standard solutions. All calibration curves showed adequate linearity in the appropriate concentration ranges with correlation coefficients >0.99 for each target compounds. The limits of detection (LODs) for these compounds were 0.02–0.5 μg·L^−1^ and the LOQs were 0.05–2.00 μg·L^−1^. The precision of method was calculated and expressed as inter-day RSD (relative standard deviation) and intraday RSD. The inter-day RSD and intraday RSD were calculated by comparing standard deviation of the peak area of standard solutions on three different days and on the same day. The inter-day RSD and intraday RSD were 3.3–7.7% and 4.4–7.5%, respectively (Table 1).

Blank matrices were spiked with standard solutions at low and high concentrations and included the d4 internal standard that was added to every sample. We then calculated the matrix factor effects to obtain IS-N MF (Internal standard normalized matrix factor) and CV (coefficient of variations) values that could be directly compared (Table 2). We found that a significant ion enhancement for imidacloprid in for cephalothorax, olefin imidacloprid in hepatopancrea, imidacloprid urea in muscle and 6-chloronicotinic acid in cephalothorax, hepatopancrea, intestine and muscle. A significant ion suppression for imidacloprid in hepatopancrea and muscle, 5-hydroxy imidacloprid in hepatopancrea, gills and intestine and olefin imidacloprid in cephalothorax, gills, intestine and muscle, imidacloprid urea in hepatopancrea and intestine. Matrix effects of all other compounds were in the normal range (0.85–1.15). Matrix matched calibration was selected for quantification of samples (Table 3).

The inter-day RSD and intraday RSD for all these analytes were calculated by comparing standard deviation of the recovery percentages of the spiked samples on three different days and the same day, respectively. The inter-day RSD and intraday RSD were all <15%. The recoveries from the *P. clarkii* matrices were all in the range of 80–112.7% (Table 4).

## 3. Real Sample Analysis

In order to determine the effectiveness of our extraction and separation method and its suitability in routine analysis, we collected 169 samples of crayfish from Qianjiang, Xiantao, Jingzhou and Xiaogan in Hubei province, and we detected imidacloprid and its metabolites. We found imidacloprid in 132 samples, 5-hydroxy imidacloprid in 12, olefin imidacloprid in 19, imidacloprid urea in 19 and 69 samples contained 6-chloronicotinic acid. These results indicated that although the parent compound imidacloprid was detected in 78% of the samples, its metabolites were also detected in 7 to 40% of these commercial *P. clarkii* samples.

## 4. Experimental

### 4.1. Reagents

Analytical standards of purity grade imidacloprid (99.8%), imidacloprid-D4 (95%), imidacloprid urea (99.7%), olefin imidacloprid (98.7%), and 6-chloronicotinic acid (99.2%) were obtained from Dr. Ehrenstorfer GmbH (Augsburg, Germany) and 5-hydroxyimidacloprid (98.0%) was obtained from Toronto Research Chemicals (Ontario, Canada). The following were obtained from the listed sources: florisil neutral alumina column (1 g/3 mL, CNW Technologies, Shanghai, China); acetic acid, acetonitrile, methanol, and ethyl acetate (Tedia, Fairfield, OH, USA); anhydrous MgSO_4_ and NaCl (Sinopharm Chemical Reagent, Beijing, China); primary secondary amine (PSA) (40–60 μm, analytical grade), C18, graphitized carbon black (GCB) (40–60 μm, analytical grade) and alumina-N sorbent (40–60 μm, analytical grade), (Bonna-Agela Technologies, Tianjin, China). Ultrapure water with a resistivity of 18.2 MΩ·cm was obtained from a Millipore filtering system (Burlington, MA, USA).

### 4.2. Standard Solutions

Imidacloprid, 5-hydroxy imidacloprid, olefin imidacloprid, imidacloprid urea and 6-chloronicotinic acid stocks were prepared in methanol at 500 mg·mL^−1^ and stored at −18 °C protected from light. Working standards were prepared the day of use in methanol/water/acetic acid (8:20:0.1) at 1, 2, 5, 10, and 20 μg·L^−1^. Imidacloprid-D4 (imidazolidin-4,4,5,5-d_4_) was used as isotope-labelled internal standard (IS) as previously described [34] and all standards and samples contained 2.0 ng·mL^−1^. Stock and working standard solutions were prepared every 3 and 1 month, respectively. The working standard solutions were used to prepare matrix-matched standards and spiked samples for validation studies.

### 4.3. Sample Preparation

*P. clarkii* plasma was extracted by inserting a 1 mL micro-injector into the heart. Animals were dissected after removal of the head. The shell was removed by lateral excisions and the remainder was defined as the cephalothorax. The yellow/brown region between the gills was removed (hepatopancreas) and the gills were then excised. The intestines were removed from the tail and the muscles were removed. All solid tissues were cut into 0.5 cm cubes and homogenized in a blender at high speed. All the samples were stored at −18 °C until analysis.

### 4.4. Sample Extraction

Tissue sample homogenates (2 g) and plasma (2 mL) were used for extraction as follows; samples were thawed at room temperature and placed in 10 mL centrifuge tubes containing 2 μL Imidacloprid-D4 internal standard solution (100 μg·L^−1^) and 3 mL acetonitrile/ 0.1% acetic acid. The tube was vortexed for 1 min and 3 mL of distilled water was added and briefly vortexed. MgSO_4_ (1.0 g) and NaCl (0.5 g) were added and the samples were immediately vortexed for 2 min. The sample was centrifuged at 5000 rpm for 5 min and the supernatant was transferred to a clean 10 mL tube. The samples were extracted again and the supernatants were combined. The combined supernatants were then passed through a neutral alumina column that was prepared by adding 0.01 g of PSA powder and 0.1 g MgSO_4_. The eluates were dried under a stream of nitrogen gas and the residue was reconstituted in 1 mL methanol: water: acetic acid (8:20:0.1) and filtered through a 0.22 μm membrane.

### 4.5. Analyte Identification

The combined high performance liquid chromatography (HPLC)—triple quadrupole mass spectrometer (MS) system TSQ Quantum Access Max equipped with LCQUAN 2.6 software (Thermo Fisher Scientific, Pittsburg, PA, USA) was used for analyte identification. Chromatographic separations utilized a Symmetry C18 column (100 × 2.1 mm, 3.5 μm) column (Waters, Burlington, MA, USA) at 35 °C. The mobile phase Component A was methanol containing 0.1% acetic acid and Component B was water containing 0.1% acetic acid. Analytes were eluted with a gradient as follows: A:B 50:50 for 2 min then 80:20 until 4 min and return to initial conditions using a flow rate of 0.3 mL·min^−1^ and an injection volume of 10 μL.

Mass spectrometry for analyte identification used a heating atmospheric electrospray ion (HESI) source in the scan mode with select response monitoring (SRM). The spray voltage was 3500 with an evaporation temperature of 250 °C. The sheath and auxiliary gases were high purity nitrogen supplied with an average pressure of 10 bar. The collision gas was high purity argon supplied at 1.5 mTorr. The temperature for the ion transfer capillary was 300 °C. The half peak width for one pole mass spectrometry scanning (Q1) was 0.7 Da and for tripolar mass spectrometry (Q3) was 0.7 Da (Table 5).

### 4.6. Validation of the Analytical Method

The validation of the HPLC-MS method was performed using the conditions recommended in the guidelines of the EU Commission Decision 2002/657/EC. After sample preparation and the detection of imidacloprid and its metabolites by HPLC-MS/MS, samples that tested pesticide-free was selected as blank matrix samples and used to construct standard curves at five concentrations. Standard curves were examined using linear regression of the ratios of chromatographic peak areas and concentration.

Matrix effects (ME) were examined by the post-extraction spiking of samples. Blank matrices were extracted from different tissues, and concentrated and dissolved with 1 mL methanol/water/acetic acid (8:20:0.1) and then spiked with analyte standard solutions. Internal standard (Imidacloprid-D4, 0.1 μg·kg^−1^) was also added per sample. The samples were analyzed and the area of the quantitative ion was compared with that obtained for the standards in solution at the same concentration levels. The specific method of calculation [35] was as follows:

Matrix factor_analytes_ = peak area_post-extracted_/peak area_pure solution_

Matrix factor_IS_ = peak area_post-extracted_/peak area_pure solution_

IS normalized matrix factor = matrix factor_analytes_/matrix factor_IS_

The limit of detection (LOD) was determined as the concentration that produced a peak area of the signal 3 × baseline and the limit of quantification (LOQ) was the concentration that produced a signal to noise ratio of 10. The detection limit of the method (MOD) refers to the lowest concentration that the signal generated by the detected substance that could be distinguished from the blank sample with 99% confidence after processing and determination including sample preparation.

Recoveries were calculated by comparing the peak areas of blank samples spiked before preparation with that of blank samples spiked after preparation in six replicates at low, middle and high concentration levels. To evaluate precision of the method, the intraday RSD values were calculated by analyzing fortified samples on the same day with the same instrument and operator. The inter-day RSD results were obtained using the identical method on three separate days with the same instrument and operator (Ning Xu et al., 2018).

## 5. Conclusions

We developed a novel modified QuEChERS-based LC-MS/MS method for the determination of pesticides in *P. clarkii*. The method provided efficient extraction and rapid cleanup of imidacloprid and its metabolites from *P. clarkii* tissues. The proposed LC-MS/MS method displayed good linearity, high sensitivity, satisfactory recoveries and precision. This method has the advantages of being fast, easy and environmentally friendly, which consumes only small quantities of reagents. The establishment of this method not only fills the blank of the standard of imidacloprid in crayfish, but also provides a theoretical basis for the control of typical pesticides in the integrated cultivation mode of rice and crayfish. Therefore, it provides reference suggestions for ensuring the consumption safety of *P. clarkii*.

## Figures and Tables

**Table 1:** Analytical performance for imidacloprid and its metabolites in solution.

Compound	Linear Range (μg·L^−1^)	Regression Equation ^a^	Correlation Coefficient (*R^2^*)	LOD ^b^ (μg·L^−1^)	LOQ ^c^ (μg·L^−1^)	RSD (%, *N* = 3)	
						Inter-Day	Intraday
IMI	0.05–2.00	Y = 0.874X + 0.079	0.9922	0.02	0.05	4.3	5.7
5-Hydroxy IMI	1.00–20.00	Y = 0.134X + 0.070	0.9947	0.50	1.00	6.8	4.4
olefin IMI	2.00–100.00	Y = 0.298X + 0.034	0.9981	0.50	2.00	5.2	5.6
IMI urea	0.10–10.00	Y = 1.631X + 0.464	0.9948	0.03	0.10	7.1	6.1
6-Chloronicotinic acid	1.00–50.00	Y = 0.436X − 0.856	0.9996	0.50	1.00	3.3	7.5

^a^ X = concentration (ng/mL), Y = counts (peak area); ^b^ Instrument detection limit (IUPAC (International Union of Pure and Applied Chemistry)criterion); ^c^ IUPAC criterion.

**Table 2:** Matrix effects of imidacloprid and metabolites in different matrixes.

Compound	Spike Level (μg·L^−1^)	Plasma		Cephalothorax		Hepatopancrea		Gill		Intestine		Muscle	
		IS-N MF	CV	IS-N MF	CV	IS-N MF	CV	IS-N MF	CV	IS-N MF	CV	IS-N MF	CV
IMI ^†^	0.1	1.055	0.09	1.752	0.05	0.695	0.08	1.023	0.05	1.103	0.06	0.359	0.06
	5.0	0.987	0.11	1.568	0.07	0.712	0.13	0.968	0.08	0.988	0.08	0.475	0.08
5-Hydroxy IMI	2.0	0.923	0.08	0.897	0.13	0.566	0.11	0.582	0.1	0.723	0.13	1.125	0.11
	100.0	0.897	0.07	0.974	0.07	0.691	0.08	0.637	0.08	0.632	0.06	0.969	0.12
olefin IMI	5.0	0.947	0.11	0.364	0.05	1.454	0.12	0.581	0.12	0.345	0.11	0.457	0.15
	200.0	0.963	0.12	0.451	0.04	1.387	0.06	0.448	0.09	0.421	0.07	0.611	0.13
IMI urea	0.2	0.996	0.05	0.865	0.1	0.503	0.07	0.999	0.04	0.538	0.05	1.325	0.13
	10.0	0.897	0.09	0.905	0.12	0.487	0.11	0.896	0.09	0.476	0.08	1.451	0.14
6-CNA ^†^	2.0	0.869	0.06	1.471	0.09	1.417	0.09	0.894	0.11	1.308	0.07	1.326	0.06
	100.0	0.857	0.14	1.302	0.06	1.349	0.05	1.057	0.14	1.411	0.07	1.259	0.15

^†^ Imidacloprid (IMI), 6-chloronicotinic acid (6-CNA).

**Table 3:** Analytical performance of imidacloprid and its metabolites in matrix-matched solutions.

Compound	Index	Plasma	Cephalothorax	Hepatopancrea	Gill	Intestine	Muscle
IMI ^†^	Linear range (μg·L^−1^)	0.1–5.0	0.1–5.0	0.1–5.0	0.1–5.0	0.1–5.0	0.1–5.0
	Regression equation ^d^	y = 0.937x + 0.395	y = 0.370x + 0.691	y = 1.478x − 0.284	y = 0.633x + 0.121	y = 0.882x + 0.109	y = 0.902x + 0.251
	Correlation coefficient (R^2^)	0.9931	0.9978	0.9969	0.9935	0.9904	0.9937
	MOQ ^e^ (μg·L^−1^ or μg·kg^−1^)	0.05	0.05	0.05	0.05	0.05	0.05
5-Hydroxy IMI	Linear range (μg·L^−1^)	2.0–100.0	2.0–100.0	2.0–100.0	2.0–100.0	2.0–100.0	2.0–100.0
	Regression equation ^d^	y = 0.119x + 0.396	y = 0.137x − 0.275	y = 0.116x + 0.703	y = 0.096x + 0.439	y = 0.082x + 0.123	y = 0.090x − 0.096
	Correlation coefficient (R^2^)	0.9977	0.9916	0.9901	0.9947	0.9996	0.9914
	MOQ ^e^ (μg·L^−1^ or μg·kg^−1^)	1.0	1.0	1.0	1.0	1.0	1.0
olefin IMI	Linear range (μg·L^−1^)	5.0–200.0	5.0–200.0	5.0–200.0	5.0–200.0	5.0–200.0	5.0–200.0
	Regression equation ^d^	y = 0.272x − 0.259	y = 0.150x − 0.043	y = 0.056x − 0.305	y = 0.400x − 2.774	y = 0.147x + 0.610	y = 0.076x + 0.090
	Correlation coefficient (R^2^)	0.9946	0.9948	0.9973	0.9942	0.9982	0.9965
	MOQ ^e^ (μg·L^−1^ or μg·kg^−1^)	2.0	2.0	2.0	2.0	2.0	2.0
IMI urea	Linear range (μg·L^−1^)	0.2–10.0	0.2–10.0	0.2–10.0	0.2–10.0	0.2–10.0	0.2–10.0
	Regression equation ^d^	y = 1.593x + 0.058	y = 2.058x + 0.194	y = 1.567x + 0.922	y = 0.723x + 4.432	y = 1.570x + 0.956	y = 0.754x + 0.948
	Correlation coefficient (R^2^)	0.9979	0.9951	0.9920	0.9984	0.9968	0.9927
	MOQ ^e^ (μg·L^−1^ or μg·kg^−1^)	0.1	0.1	0.1	0.1	0.1	0.1
6-CNA ^†^	Linear range (μg·L^−1^)	2.0–100.0	2.0–100.0	2.0–100.0	2.0–100.0	2.0–100.0	2.0–100.0
	Regression equation ^d^	y = 0.225x + 1.364	y = 0.528x + 0.576	y = 0.571x + 0.143	y = 0.236x + 1.269	y = 0.417x + 2.456	y = 0.542x + 0.954
	Correlation coefficient (R^2^)	0.9934	0.9947	0.9963	0.9981	0.9976	0.9935
	MOQ ^e^ (μg·L^−1^ or μg·kg^−1^)	1.0	1.0	1.0	1.0	1.0	1.0

^†^ Imidacloprid (IMI), 6 chloro-nicotinic acid (6-CNA); ^d^ x = concentration (ng/mL), y = counts (peak area); ^e^ EURACHEM (Europe chemical organisation) criterion (RSD 10%).

**Table 4:** Recoveries and RSDs from spiked samples ^‡^.

Compound	Spike level (μg kg^−1^)	Plasma			Cephalothorax			Hepatopancrea			Gill			Intestine			Muscle		
		Recovery (%)	Intraday RSD	Inter-days RSD	Recovery (%)	Intraday RSD	Inter-days RSD	Recovery (%)	Intraday RSD	Inter-days RSD	Recovery (%)	Intraday RSD	Inter-days RSD	Recovery (%)	Intraday RSD	Inter-days RSD	Recovery (%)	Intraday RSD	Inter-days RSD
IMI	0.1	89.6	7.2	5.6	96.3	6.3	6.8	102.2	9.6	6.5	91.6	9.8	6.4	100.5	8.1	10.5	106.3	4.6	8.0
	1	97.2	9.5	6.9	87.4	5.5	7.8	97.6	7.3	5.9	86.4	7.7	7.5	96.8	5.9	8.6	110.4	8.6	7.7
	5	102.3	4.3	8.2	106.7	9.8	9.4	98.3	5.2	8.1	88.9	6.1	8.6	94.1	4.2	9.4	112.7	9.1	6.4
5-Hydroxy IMI	2.0	89.6	6.0	5.4	107.6	8.1	10.4	89.6	6.8	6.3	97.6	5.0	9.1	103.8	6.8	7.9	94.6	7.6	5.2
	20	85.1	8.5	6.3	99.8	9.5	12.3	83.4	6.1	8.4	96.3	9.1	11.0	85.6	9.9	5.7	84.1	5.4	9.3
	100	97.7	9.7	8.0	110.7	11.5	8.4	91.2	7.8	9.5	102.6	6.5	7.3	107.6	10.5	6.1	86.9	6.9	4.6
olefin IMI	5.0	88.7	10.6	4.6	97.2	7.6	7.6	85.2	9.5	7.3	110.1	4.6	6.9	96.8	8.6	8.9	88.7	7.6	8.2
	50.0	90.3	5.8	7.6	88.7	6.8	9.4	86.8	6.4	6.9	95.4	8.6	8..8	94.6	7.4	9.9	94.2	9.3	9.4
	200.0	100.5	6.4	6.8	95.4	8.3	8.3	91.6	3.7	9.5	86.3	9.2	9.1	82.6	9.9	6.8	90.5	8.2	7.6
IMI urea	0.2	98.6	7.2	7.7	86.7	9.4	9.4	86.7	7.9	10.9	91.5	7.7	10.6	86.4	5.8	12.5	101.4	8.6	9.5
	2.0	91.8	9.1	8.0	89.8	6.6	6.3	81.1	5.1	12.6	86.8	8.5	8.3	89.1	6.8	7.8	90.6	7.2	4.3
	10.0	81.5	8.8	9.6	92.5	11.8	8.5	80.6	5.2	9.4	97.1	4.9	9.1	102.2	7.1	9.3	80.4	6.8	9.8
6-CNA ^†^	2.0	87.1	6.9	5.1	103.3	10.6	10.5	97.6	9.8	8.2	95.6	8.2	7.6	87.6	8.6	8.6	99.6	9.4	8.6
	20.0	80.9	8.3	8.5	88.6	9.1	11.9	99.7	8.8	7.3	85.6	5.8	12.1	91.7	9.8	10.0	89.4	12.6	5.5
	100.0	89.7	11.2	5.8	97.4	8.3	10.6	105.9	7.3	10.6	97.7	11.9	5.5	90.5	12.2	9.3	100.4	11.3	6.7

^†^ 6-Chloro-nicotinic acid (6-CNA); ^‡^ *n* = 6.

**Table 5:** MS parameters for imidacloprid and its metabolites.

Analyte	Ionization Mode	Precursor Ion (*m*/*z*)	Product Ion (*m*/*z*)	Collision Energy (eV)
Imidacloprid	positive	256.0	208.8/174.9	16/19
Imidacloprid-D4	positive	260	179/213	19/19
5-Hydroxy Imidacloprid	positive	272.0	225/226.1	14/9
Olefin imidacloprid	negative	251.9	204.9/81.1	14/10
Imidacloprid urea	positive	212.0	126/128	24/18
6-Chloronicotinic acid	negative	156.1	112/35.1	13/26

## Data Availability

The data presented in this study are available on request from the corresponding author.

## Sample Availability

Samples of the compounds are available from the authors.
